# Penile Calciphylaxis: A Successfully Treated Case

**DOI:** 10.7759/cureus.54824

**Published:** 2024-02-24

**Authors:** Narine Misakyan, Amer Abu-Shanab, Shazia Shah

**Affiliations:** 1 Internal Medicine, Capital Health Medical Center, Trenton, USA; 2 Internal Medicine, Monmouth Medical Center, Long Branch, USA

**Keywords:** penile calciphylaxis, sodium thiosulfate, end stage renal disease (esrd), types 2 diabetes, ischemia

## Abstract

Penile calciphylaxis is a rare presentation of calcific uremic arteriolopathy and can be a life-threatening condition usually seen in patients with end-stage renal failure with hemodialysis. The clear etiopathogenesis of calciphylaxis is not fully understood, but it is postulated to be characterized by the accumulation of calcium in the microvessels of adipose tissue and skin, which leads to ischemia and necrosis, causing painful ulcerations, and could potentially be complicated by sepsis and mortality. End-stage renal disease (ESRD) is one of the major risk factors for penile calciphylaxis. In this report, we describe a case of a 53-year-old Hispanic male patient with ESRD and diabetes on hemodialysis, who presented with a five-day history of acute, severe, burning, non-radiating pain to the head of his penis associated with black discoloration. He was diagnosed with penile calciphylaxis and received a combination of conservative and surgical interventions, resulting in a highly positive outcome marked by complete healing of the scar without any reported complications.

## Introduction

Penile calciphylaxis, a form of calcific uremic arteriolopathy, poses a potential life-threatening risk and is observed in around 6% of individuals with end-stage renal disease (ESRD) undergoing hemodialysis [1]. The clear etiopathogenesis of calciphylaxis is not fully understood, but it is postulated to be characterized by the accumulation of calcium in the microvessels of adipose tissue and skin, which may lead to ischemia and necrosis, causing painful ulcerations. It could potentially be complicated by sepsis and mortality [1-3].

ESRD stands out as a significant contributor to the risk of penile calciphylaxis. Additional factors that elevate this risk encompass diabetes mellitus, obesity, hyperphosphatemia, hyperparathyroidism, specific medications, hypercoagulable states, and belonging to Caucasian ethnicity [1,2]. The mortality rate is notably elevated in individuals with penile calciphylaxis due to their susceptibility to infections and the development of dry gangrene [2,3]. The literature currently documents a limited number of penile calciphylaxis cases in patients with either ESRD or diabetes mellitus (DM). In this report, we describe a case of penile calciphylaxis who received a combination of conservative and surgical interventions, resulting in a highly positive outcome.

## Case presentation

A 53-year-old Hispanic male with a past medical history of ESRD on hemodialysis presented to the emergency department complaining of a five-day history of acute, severe, burning, non-radiating pain to the head of his penis associated with black discoloration. He denied any traumatic history of his penis or recent sexual activities. He denied fever and chills but had admitted multiple episodes of vomiting preceding the penile pain. As per the patient, he did not pass urine for the past four days. The patient also described intermittent claudication of both legs for the past couple of months. The rest of his past medical history includes type 2 DM, hypertension, ischemic stroke with right hemiparesis, and baseline erectile dysfunction, and he was on metformin, metoprolol, nifedipine, hydralazine, atorvastatin, aspirin, sevelamer, and multivitamins.

**Figure 1:**
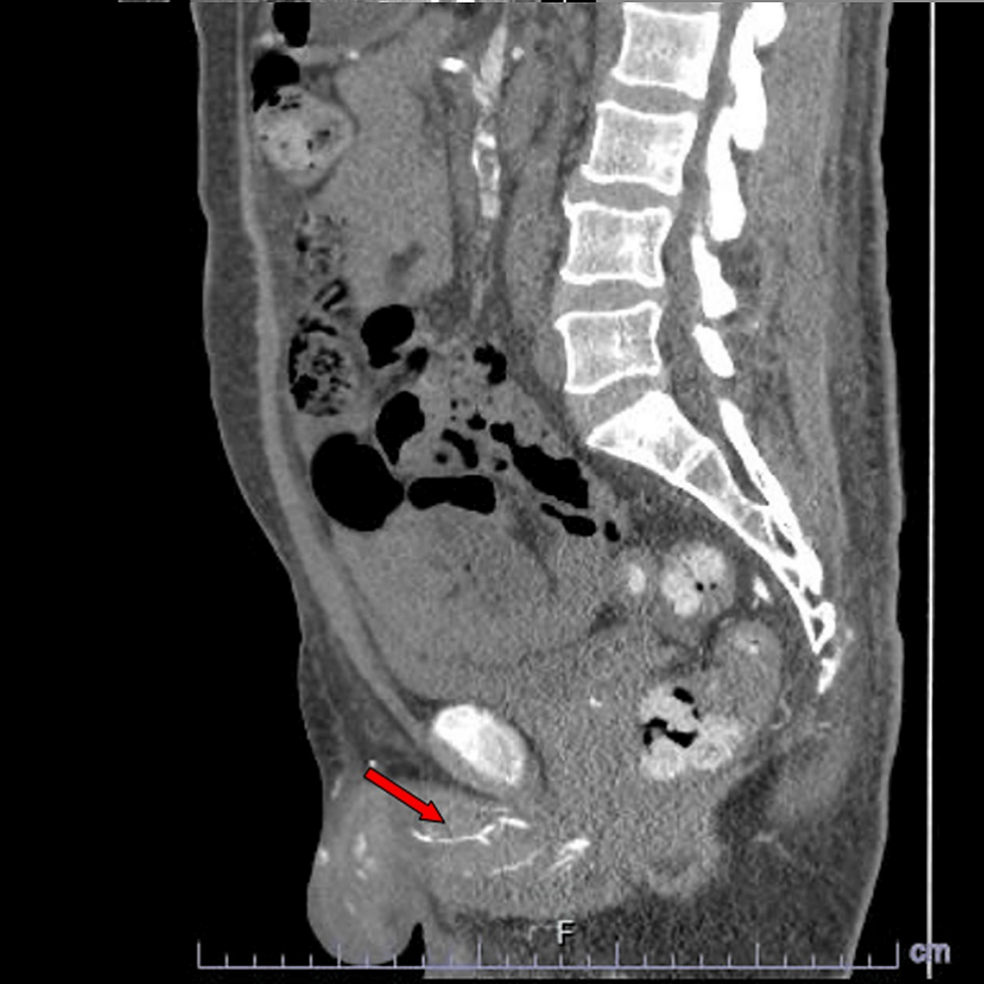
Pelvis CT scan showing diffuse calcification of penile vessels

On examination, vital signs were stable. The patient was alert and oriented. Genitourinary examination revealed a well-demarcated, black, uncircumcised foreskin unable to retract, with malodorous and purulent discharge from the penile glans. Laboratory analysis was significant for leukocytosis 25.8 μl, hyperphosphatemia 6.0 mg/dl, and high parathyroid hormone (PTH) of 100 pg/ml. A CT scan of the abdomen and pelvis demonstrated air around the tip of the penis, which, in the setting of an uncircumcised penis, likely represents gangrenous soft tissue gas with calcified penile vessels (Figure 1). There were also described concentric calcific plaques involving almost all the sub-branches of the celiac axis, superior mesenteric, and bilateral renal arteries. Blood cultures were negative.

Based on clinical, laboratory, and radiologic findings, the diagnosis of penile calciphylaxis was raised, thus, the patient was initiated on broad-spectrum antibiotics, including vancomycin, metronidazole, and cefepime, and culture was sent from the blood and the gangrenous site. The urology service was consulted for surgical intervention. The patient underwent a partial penectomy. Surgical histopathology revealed extensive gangrenous necrosis and severe acute inflammation of the penile glans, shaft, and skin. Few calcified blood vessels were also noted. Both cultures continued to be negative for growth, thus the patient was continued on cefepime only for an additional two weeks and was discharged on postoperative day 18. The patient was also started on treatment with sodium thiosulfate thrice weekly on hemodialysis days. The patient tolerated treatment without side effects. The patient regularly followed up with nephrology after the discharge with no significant postoperative complications documented.

## Discussion

Penile calciphylaxis is a multifactorial and rare presentation of systemic calciphylaxis associated with high mortality [1-3]. The high mortality rate is predominantly from superimposed sepsis [1,2]. Multiple studies have been done to identify the possible causes of calciphylaxis, but they are limited by sample size. Some of the risk factors established are end-stage renal failure, dialysis, diabetes, hypercalcemia, hypoalbuminemia, warfarin therapy, disturbances in serum calcium-phosphate metabolism, and Caucasian ethnicity [1-4]. In our case, the patient is a male and belongs to the Hispanic ethnicity.

In previously reported cases, more than half of the patients with penile calciphylaxis had underlying diabetes. In contrast, less than 50% of patients with penile calciphylaxis had ESRD. That data suggests that diabetes is one of the most common predisposing factors [5]. In our case, the presented patient had both diabetes and was on hemodialysis for ERDS, which significantly increased his risk of developing penile calciphylaxis as suggested by Ohta et al. [5].

Although calciphylaxis has a complex pathophysiology that is still not fully understood, hemodialysis seems to play a role in altering the pro- and anti-calcification factors that favor calcification of the medial layer of small arteries, which is believed to be the central mechanism for developing calciphylaxis [6]. Calcium and phosphate levels are increased due to their impaired metabolism in patients with chronic kidney disease. An increase in the calcium-phosphate product increases the risk of vascular ossification [5-7]. Chronic renal failure can result in low levels of fetuin-A, which is believed to play a role in preventing vascular ossification calcification. Vascular smooth muscle cells differentiate into an osteochondrogenic phenotype, which leads to the production of calcium phosphate nanocrystals and calcification. Endothelial cells undergo intimal hyperplasia along with varying degrees of necrosis. Endothelial necrosis then leads to vascular thrombosis. All these events eventually cause luminal obstruction and tissue infarction with developing painful black necrotic eschars [5-7]. In our patient, extensive gangrenous necrosis and severe acute inflammation of the penile glans, shaft, and skin with few calcified blood vessels were found in histopathology. No endothelial hyperplasia was found.

The diagnosis can be made in a combination of the clinical picture, blood work, biopsy, and radiological findings as in this case (Figure 1) since early diagnosis may favor preventing the progression of the disease and choosing a less invasive approach for the treatment of the penile calciphylaxis [4,6]. Penile Doppler ultrasound may be performed to assess vascular patency and blood flow within the penile vessels [4]. CT scan is the most sensitive tool to assess the extent of calcifications. A CT with contrast can appreciate tissue ischemia or the presence of air. MRI is also useful to assess the extent of ischemia and is more favorable in patients with CKD to avoid the exposure of the contrast [4].

Treatment of penile calciphylaxis requires a multi-disciplinary approach involving nephrology, urology, dermatology, plastic surgery, wound care, pain management, and correcting underlying laboratory abnormalities [1,2,6]. There is some consensus that sodium thiosulfate can be used as a chelating agent that helps in altering the mechanism and preventing the propagation of calciphylaxis but the mechanism is still not fully understood [2]. It was used in our patient with very good results in combination with surgery. Surgical options include partial or total penectomy depending on the individual case [1,2]. In our patient, and as per urology experience, the best option for our case was partial penectomy, which was done successfully with no complications or need for further surgical interventions. We also need to correct underlying abnormalities like the serum levels of parathyroid hormone. In hyperparathyroidism such as in our case, total or partial parathyroidectomy may be one of the options to control symptoms though due to the lack of strong evidence to improve the overall outcome, it was not done in our patient, as he was not a candidate for it [2]. Cinacalcet is also being used to suppress PTH levels with downstream effects on calcium and phosphorus. Prevention of systemic infection is also vital [8].

## Conclusions

Penile calciphylaxis represents a significant complication observed in individuals dealing with diabetes and chronic renal failure undergoing hemodialysis. Timely detection and effective management play a pivotal role in achieving favorable outcomes. Employing a comprehensive approach involving medical interventions, such as sodium thiosulfate and antibiotics, along with surgical measures like amputation of the gangrenous part of the penis, can lead to positive results. It is imperative to promote awareness among patients with ESRD and diabetes mellitus undergoing hemodialysis, emphasizing the recognition of signs associated with calciphylaxis. Encouraging patients to promptly communicate any emerging symptoms to their primary care physicians or nephrologists, or even attending the emergency department, is essential, as early intervention significantly contributes to preventing adverse prognoses.
